# Supplementary material for: A Likelihood Approach for Real-Time Calibration of Stochastic Compartmental Epidemic Models
Source: PLoS Comput Biol. 2017 Jan 17;13(1):e1005257. doi: 10.1371/journal.pcbi.1005257 (PMC5240920; doi:10.1371/journal.pcbi.1005257)
Supplement: S1 File — (TAR.GZ) [file pcbi.1005257.s014.tar.gz › HSPH_Online-SI-Revision/Additions/EnKF08/n100-extreme-SEITR_noisy/n100-extreme-SEITR_noisy_PF_EnKF-summery.pdf]

peak -8	R0	$1/\Theta_2$	inf	inflog	newly	newlylog	Reff	AttackRate
mean	44.4	54.5	71 555.1	2.1	103 574.	2.2	55.6	57.8
median	40.4	53.4	21 644.8	2.2	26 308.2	2.3	53.8	52.7
Quantile .1	17.8	38.4	470.	0.6	709.5	0.7	36.9	34.8
Quantile .9	72.5	73.	192 679.	3.1	378 277.	3.3	76.6	86.
44	27	40						
length	50							

peak -4	R0	$1/\Theta_2$	inf	inflog	newly	newlylog	Reff	AttackRate
mean	45.	58.9	510.4	0.7	388.7	0.7	70.	82.6
median	40.3	62.2	104.9	0.7	140.4	0.7	71.4	82.2
Quantile .1	17.5	39.5	76.4	0.4	78.1	0.4	53.4	62.9
Quantile .9	88.6	73.3	1459.4	1.1	892.6	0.9	83.8	98.7
40	19	16						
length	50							

peak	R0	$1/\Theta_2$	inf	inflog	newly	newlylog	Reff	AttackRate
mean	45.8	49.8	86.7	1.1	90.3	0.9	162.	97.6
median	36.7	49.8	86.1	1.	90.4	0.8	141.6	95.8
Quantile .1	21.6	29.6	80.	0.7	79.4	0.6	61.3	77.6
Quantile .9	85.9	69.1	94.6	1.5	102.	1.3	291.7	117.4
40	18	0						
length	50							

peak +4	R0	$1/\Theta_2$	inf	inflog	newly	newlylog	Reff	AttackRate
mean	52.8	58.9	119.9	0.4	239.2	0.4	1060.5	96.1
median	48.2	61.7	89.6	0.4	204.1	0.4	732.9	93.4
Quantile .1	21.8	39.7	62.4	0.3	79.3	0.3	293.	77.3
Quantile .9	85.3	75.8	195.1	0.7	412.2	0.6	2049.1	123.4
41	17	0						
length	50							

peak +8	R0	$1/\Theta_2$	inf	inflog	newly	newlylog	Reff	AttackRate
mean	47.1	50.4	1532.7	0.7	2776.8	0.9	1778.3	89.8
median	40.6	46.9	385.8	0.6	1124.5	0.8	1328.7	88.4
Quantile .1	21.	27.9	95.6	0.3	287.	0.5	363.9	74.
Quantile .9	81.	73.	3231.4	1.2	7934.8	1.4	3596.	105.1
37	19	0						
length	50							
